# Role of Matrix Metalloproteinases and Their Inhibitors in Locally Invasive Papillary Thyroid Cancer

**DOI:** 10.3390/biomedicines10123178

**Published:** 2022-12-08

**Authors:** Irena Ivković, Zgjim Limani, Antonia Jakovčević, Dražen Huić, Drago Prgomet

**Affiliations:** 1 Department of Otorhinolaryngology-Head & Neck Surgery, School of Medicine Zagreb, University Hospital Centre Zagreb, 10 000 Zagreb, Croatia; 2 Department of ENT-Head & Neck Surgery, University Clinical Center of Kosovo, 10 000 Prishtina, Kosovo; 3 Faculty of Medicine, University of Prishtina “Hasan Prishtina”, 10 000 Prishtina, Kosovo; 4 Department of Pathology and Cytology, University Hospital Centre Zagreb, 10 000 Zagreb, Croatia; 5 Department of Nuclear Medicine and Radiation Protection, School of Medicine Zagreb, University Hospital Centre Zagreb, 10 000 Zagreb, Croatia

**Keywords:** matrix metalloproteinase, MMP-1, MMP-2, MMP-9, TIMP-1, TIMP-2, papillary invasive carcinoma, thyroid gland

## Abstract

Locally invasive papillary thyroid carcinoma (PTC) protrudes beyond the thyroid capsule and invades local structures. Matrix metalloproteinases (MMPs) and their inhibitors (TIMPs) are implicated in local invasion and metastasis in PTC. The aim of our study was to determine expression levels of MMP-1, MMP-2, MMP-9, TIMP-1, and TIMP-2 in tissue specimens of invasive and non-invasive PTC. Our hypothesis was that expression levels of these biomarkers correlate with the development of locally invasive PTC. In our single-center study we retrospectively investigated MMP and TIMP expression levels in 50 samples of thyroid tissue diagnosed as locally invasive papillary carcinoma (study group) and 30 samples of thyroid tissue diagnosed as non-invasive, non-metastatic papillary carcinoma (control group). Tissue specimens were immunohistochemically stained with primary monoclonal antibodies against MMP-1, MMP-2, MMP-9, TIMP-1, and TIMP-2. When correlating expression levels of MMPs and TIMPs in thyroid tissue, statistically significant differences were found for MMP-1 and TIMP-1 expression (*p* < 0.001; Mann–Whitney U test) with the highest levels of expression in the invasive PTC group. Although expression of MMP-9 and TIMP-2 was higher in invasive PTC, the differences were not statistically significant. Elevated expression of MMP-1 and TIMP-1 in tumor tissue can predict invasiveness for PTC.

## 1. Introduction

Matrix metalloproteinases (MMPs) are a large family of calcium-dependent zinc-containing endopeptidases that are responsible for tissue remodeling and degradation of the extracellular matrix (ECM) contents, including collagens, elastins, gelatin, matrix glycoproteins, and proteoglycans [1,2]. MMPs are excreted by fibroblasts, osteoblasts, endothelial cells, macrophages, neutrophils, and lymphocytes. There are at least 26 human MMPs recognized. On the basis of their specific substrates, MMPs are classified into collagenases, gelatinases, stromelysins, and matrilysins [1,2]. MMP-1 belongs to the collagenases whereas MMP-2 and MMP-9 are classified as gelatinases. MMP-2 is classified into the gelatinase subgroup because of its domain composition, even though it has collagenolytic activity as well (digests collagens I, II, and III) [1,2]. Under physiological conditions, the proteolytic activity of MMPs is controlled at any of the following three known stages: activation of the zymogens, transcription, and inhibition of the active forms by various tissue inhibitors of MMPs (TIMPs) [3]. Tissue inhibitors of metalloproteinases (TIMPs) are important regulators of MMP activity. The TIMP family consists of TIMP-1, TIMP-2, TIMP-3, and TIMP-4 [3,4,5]. TIMPs have been shown to bind to the proenzyme forms of MMP-2 and MMP-9 with a high degree of specificity. TIMP-1 binds to pro-MMP-9, whereas TIMP-2 is more widely distributed and binds to all MMPs with a higher specificity for MMP-2 [5,6,7]. In resting states of physiological conditions in adult tissues MMPs are minimally expressed [8]. Their expression is regulated by hormones, growth factors, and cytokines. In pathological conditions this equilibrium is shifted toward increased MMP activity leading to tissue degradation. Over-expression of MMPs results in an imbalance between the activity of MMPs and TIMPs that can lead to numerous pathological disorders [3]. During tumorigenesis, tumor cells, through the activation of MMPs, degrade the basement membrane and the extracellular matrix components and invade the surrounding stroma [3]. MMPs also play an important role in apoptosis with both apoptotic and anti-apoptotic action [9,10]. Among MMPs, MMP-1, MMP-2, and MMP-9 are reported to be most commonly related to malignant tumors, their metastases, and local invasion [11]. Overexpression of MMPs in tumor tissue has been reported in many types of solid tumors, including breast, ovary, colorectal, and pancreatic carcinomas, squamous carcinomas of the head and neck, and other carcinomas [12,13,14,15,16,17,18]. Human thyroid carcinoma tissue has also been reported to express MMP-1, MMP-2, and MMP-9 and these MMPs were localized in tumor cells and/or in the fibroblasts adjacent to or close to the invading tumor cells [19,20,21,22,23,24,25,26,27,28,29,30,31,32]. TIMPs, as inhibitors of MMPs, were initially considered as potential inhibitors of invasion and metastases that could alter the metastatic potential of cancer cells [33]. Many studies have reported that TIMPs can act independently of MMPs. Moreover, TIMP-1 is reported to promote tumorigenesis by stimulating cell proliferation and growth and through its anti-apoptotic function [20,28,30,34,35,36,37]. In contrast TIMP-2 is reported to suppress tumor growth and metastasis [38,39]. Locally invasive papillary thyroid carcinoma (PTC) is a cancer that by definition protrudes beyond the thyroid capsule and invades local structures causing significant morbidity and mortality [40,41]. It comprises between seven and 15% of all cases of thyroid cancer [40]. Invasive PTC is characterized by an increased male to female ratio and its incidence is reported to increase with age [42]. Clinically it may present as a hard, fixed neck mass with neck pain and stiffness, dysphonia, voice weakness, fatigue, hemoptysis, stridor, dyspnea, and dysphagia. Surgical resection is the treatment of choice for patients with locally invasive PTC in combination with adjuvant therapy—radioactive iodine and external beam radiotherapy [40,41,43]. The extent of treatment in these patients is determined by the structures that are invaded. The most-commonly invaded structures are the strap muscles, recurrent laryngeal nerve, larynx, trachea, and pharynx [40,41,43]. Proper management of the central neck compartment plays an important role in the treatment of these cases as well [40].

Numerous studies have reported MMP-1, MMP-2, MMP-9, and TIMP-1 to correlate with PTC invasion and metastases [19,20,21,22,23,24,25,26,27,28,29,30,31,32,33,34,35,36,37]. The objective of our study was to examine the expression of tumor-progression-associated MMPs (MMP-1, MMP-2, and MMP-9) and tissue inhibitors of metalloproteinases (TIMP-1 and TIMP-2) in locally invasive PTC and their relation to clinicopathological features. To the best of our knowledge, correlation between expression of matrix metalloproteinases (MMP-1, MMP-2, and MMP-9) and tissue inhibitors of metalloproteinases (TIMP-1 and TIMP-2) in locally invasive PTC has never been evaluated. Our hypothesis was that expression of MMPs (MMP-1, MMP-2, and MMP-9) and TIMPs (TIMP-1 and TIMP-2) were related to invasiveness of PTC. The availability of a potential marker for invasiveness could have important implications in tailoring therapy, given the morbidity associated with recurrent neck surgeries and external radiation therapy. It could also raise the index of suspicion for occult metastatic disease at presentation, indicating the need for neck dissection in high-risk patients.

## 2. Patients and Methods

### 2.1. Participants

Tissue samples of non-invasive PTC and locally invasive PTC were obtained from the archives of the Clinical Institute of Pathology, Zagreb University Hospital Centre (Zagreb UHC), collected from patients operated on at the Departments of ENT and Head and Neck Surgery, Zagreb UHC, between 25 February 2000 and 3 January 2016. All patients were operated on by the same surgeon and had undergone preoperative ultrasound examination and fine-needle aspiration biopsy of suspicious thyroid nodules and lymph nodes to determine the extent of the surgery. In total, samples of 80 patients were analyzed. Total thyroidectomy was performed on all patients, whereas patients with confirmed metastasis underwent selective neck dissection of either central (level VI) or lateral neck (level II-V). Subjects were divided into two groups as follows: Group 1—control group of 30 patients with non-invasive PTC; Group 2—study group of 50 patients with invasive PTC that protruded beyond the thyroid capsule and invaded local structures such as the strap muscles, recurrent laryngeal nerve, larynx, trachea, and pharynx.

### 2.2. Hematoxylin-Eosin Staining

Tissue samples were fixed for 24 h in 10% buffered formalin immediately after resection, dehydrated in ethanol, embedded in paraffin, cut into 5 µm thin sections, and stained with a standard method (hematoxylin-eosin, H&E) for light microscopy analysis. Representative tumor tissue and adjacent non-tumorous thyroid tissue were defined and marked on H&E slides for further immunohistochemical analysis. Histopathology and immunohistology of PTC specimens were reviewed by two experienced pathologists.

### 2.3. Immunohistochemistry

**Figure 1:**
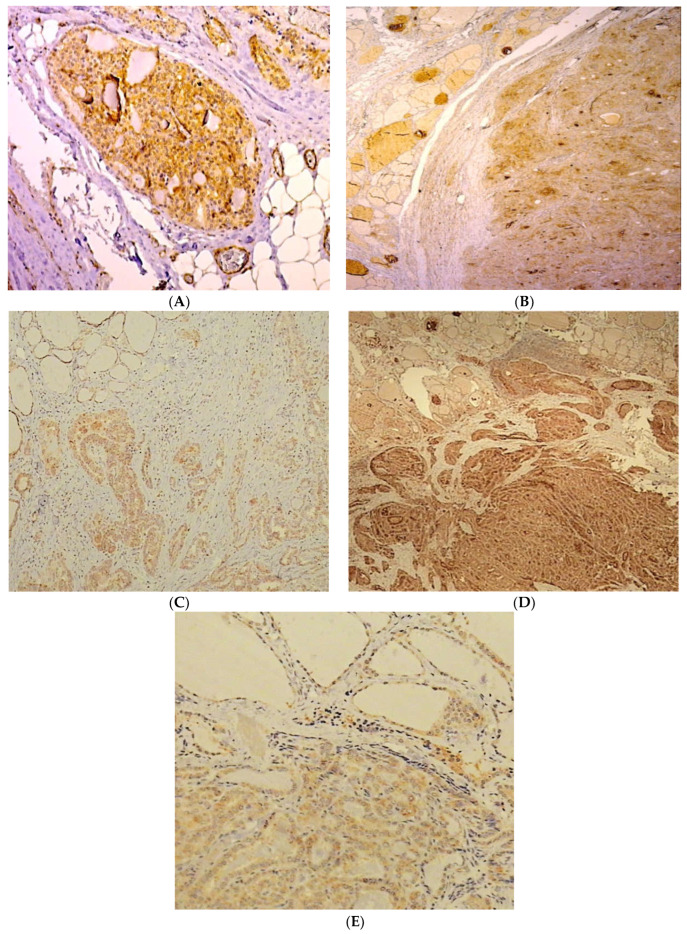
(**A**) Immunohistochemical expression of MMP-1 in invasive PTC. Tumor cells show strong positivity in the cytoplasm, ×200; (**B**) Immunohistochemical expression of MMP-2 in PTC. Tumor cells show strong positivity in the cytoplasm (left) and weak cytoplasmic expression in the adjacent non-tumor cells (right), ×25; (**C**) Immunohistochemical expression of MMP-9 in invasive PTC. Weak to moderate positivity is shown in the cytoplasm of tumor cells (left) and the adjacent non-tumor cells (right), ×200; (**D**) Immunohistochemical expression of TIMP-1 in invasive PTC. Tumor cells show strong positivity in the cytoplasm (left), while adjacent non-tumorous tissue is also positive but at low intensity (right), ×25; (**E**) Immunohistochemical expression of TIMP-2 in invasive PTC. Tumor tissue shows weak, positive intensity in the cytoplasm (left) and the adjacent non-tumorous tissue (right), ×200.

From the selected paraffin blocks, 3 to 4 µm thick sections were obtained for immunohistochemical (IHC) staining. Primary monoclonal antibodies against MMP-1 (ab52631, clone EP1247Y, dilution 1:50, Abcam, Cambridge, UK), MMP-2 (NCL-MMP2-507, clone 17B11, dilution 1:75, Novocastra, Leica Biosystems, Nussloch, Germany), MMP-9 (NCL-MMP9-439, clone 15W2, dilution 1:80, Novocastra, Leica Biosystems, Nussloch, Germany), TIMP-1 (M7293, clone VT7, dilution 1:100, Dako, Glostrup, Denmark), and TIMP-2 (NCL-TIMP2-487, clone 46E5, dilution 1:100, Novocastra, Leica Biosystems, Nussloch, Germany) were applied using the immunoperoxidase avidin–biotin method in an automatic stainer (Autostainer, Dako, Glostrup, Denmark). IHC analysis was performed with the EnVision™FLEX (K800 Dako, Glostrup, Denmark) detection system. Cervix squamous cell carcinoma was used as positive control for MMP-1, ulcerative colitis (colon) for MMP-2, liver for MMP-9, colorectal adenocarcinoma for TIMP-1, and placenta for TIMP-2 as per the manufacturer’s instructions. Negative controls were the same tissue, avoiding the primary antibody. Positive staining for all primary antibodies was defined as cytoplasmic staining in cells. All slides were analyzed using light microscopy. MMP-2, MMP-9, MMP-1, TIMP-1, and TIMP-2 immunostaining in the tumor and adjacent non-tumorous thyroid tissue were evaluated semi-quantitatively as follows: 0, negative; 1+, weak; 2+, moderate; 3+, strong (Figure 1).

Informed consent was obtained and the Institutional Review Board approved this study under the Helsinki Declaration guidelines, most recently amended in 2013.

#### Statistical Methods

Continuous data distributions were assessed for normality with Kolmogorov-Smirnov tests and according to the results appropriate non-parametric tests were used in the followed analyses. Differences in categorical variables were assessed with Fisher–Freeman–Halton’s exact tests while Mann–Whitney U tests were used for analysis of continuous variables between invasive and non-invasive tumor groups. Spearman correlation coefficients (rho) were used to assess correlations between enzyme expression in tumor tissue in invasive and non-invasive tumor types. All significant variables on bivariate levels were used as predictor variables in a binary logistic regression for multivariate prediction of belonging to the invasive tumor group. MedCalc^®^ Statistical Software version 20.116 (MedCalc Software Ltd., Ostend, Belgium; https://www.medcalc.org; accessed on 7 November 2022) was used in all statistical procedures. All *p* values below 0.05 were considered significant.

## 3. Results

A total of 80 subjects was included in the study, divided into two groups—50 subjects with locally invasive and 30 subjects with non-invasive PTC.

There were no significant differences between invasive and non-invasive tumor groups regarding histology type, T status, and patient gender distribution (Table 1).

Patients with invasive tumors were significantly younger with median (IQR) 45.0 (30.8–54.3) years versus 54.0 (41.8–62.3) years, *p* = 0.027. The invasive tumor group also had significantly larger tumor size (1.2 (1.0–2.0) cm vs. 1.0 (0.6–1.5) cm; *p* = 0.018), increased MMP-1 expression in tumor cells (80.0% (67.5–90.0%) vs. 70.0% (45.0–85.0%); *p* = 0.049), increased TIMP-1 expression in tumor cells (95.0% (85.0–95.0%) vs. 82.5% (50.0–90.0%); *p* < 0.001), increased MMP-1 expression in adjacent tissue cells (60.0% (50.0–80.0%) vs. 50.0% (37.5–65.0%); *p* = 0.029), increased MMP-9 expression in adjacent non-tumor tissue cells (82.5% (67.5–90.0%) vs. 70.0% (50.0–90.0%); *p* = 0.017), and significantly increased expression of TIMP-1 in the adjacent tissue cells (80.0% (67.5–90.0%) vs. 50.0% (23.8–70.0%); *p* < 0.001) (Table 2). All these variables were used in the multivariate regression model (Table 3).

The regression model was statistically significant (*p* = 0.001), explaining 36.4% of dependent variable variance (R^2^) and correctly classifying 78.8% of subjects. In multivariate surroundings only two predictor variables were significant for belonging to the invasive tumor group: increased TIMP-1 expression in the adjacent tissue with odds ratio (OR) of 3.97 (95% confidence interval (CI): 1.42–11.13; *p* = 0.009) and larger tumor size with OR of 1.81 (95% CI: 1.01–3.25; *p* = 0.048) (Table 3).

MMP-1, MMP-2, MMP-9, TIMP-1, and TIMP-2 expression correlations between tumor tissue in invasive and non-invasive PTC are shown in Table 4. In the non-invasive tumor group there was only one significant correlation, between TIMP-2 and MMP-2 expression (rho = 0.378, *p* = 0.039). In the invasive tumor group there were two significant correlations: first between MMP-1and MMP-2 expression (rho = 0.306, *p* = 0.031) and second between TIMP-2 and MMP-9 expression (rho = 0.464, *p* = 0.001). All correlations were positive.

## 4. Discussion

A significant increase in the incidence of PTC worldwide [42,44,45], and the excellent overall prognosis has focused clinical interest on finding potential biomarkers that could detect the small percentage of patients who are at increased risk for an unfavorable course of the disease such as invasion of surrounding structures, development of occult and clinical metastases, local/locoregional recurrence, and hematogenous dissemination of the disease. The frequency of this unfavorable group of PTC is reported to increase with age [42], although, in our study, subjects with invasive tumors were significantly younger with median (IQR) 45.0 (30.8–54.3) years versus 54.0 (41.8–62.3) years, *p* = 0.027.

Moreover, an increased male-to-female ratio in patients with locally invasive PTC is reported in the literature [44,45]. Our study confirms a higher percentage of women in the control group with non-invasive PTC; however, the groups were borderline comparable, and this could be due to the small sample size and the rare occurrence of locally invasive PTC.

Numerous studies have reported that MMP-1, MMP-2, and MMP-9 enzymes and TIMP-1 and TIMP-2 inhibitors play an active role in angiogenesis, local invasiveness, and metastatic potential of malignant tumors [12,13,14,15,16,17,18,19,20,21,22,23,24,25,26,27,28,29,30,31,32,33,34,35,36,37,38,39]. In our study, we assessed and correlated the immunohistochemical expression of MMP-1, MMP-2, MMP-9, TIMP-1, and TIMP-2 in tissue specimens of locally invasive and non-invasive PTC. The expression of all enzymes and their inhibitors was increased in invasive PTC compared to non-invasive ones, except for MMP-2 for which we did not encounter a noteworthy difference in expression between invasive and non-invasive PTC.

Up-regulated MMP-1 was found to be positively related to aggressive clinical features, worse survival, extracellular matrix-related pathways, oncogenic immune microenvironment, mutations, stemness, and dedifferentiation in PTC in a recent study reported by Zhou et al. using transcriptome data analysis of poorly differentiated cancer/anaplastic cancer and PTC from the Gene Expression Omnibus and the Cancer Genome Atlas databases [19]. Bumber et al. reported significantly higher immunohistochemical expression of MMP-1 (Kruskal–Wallis, *p* = 0.020) and TIMP-1 (Kruskal–Wallis, *p* < 0.001) in patients with cervical lymph node metastases compared to patients without metastases in PTC [20]. They reported the highest expression in patients with metastasis in the lateral regions of the neck and concluded that increased expression of MMP-1 and TIMP-1 in tumor tissue of patients with PTC can be considered a predictive factor for the development of metastases, particularly for the development of metastases in lateral regions of the neck [20]. Kameyama et al., in an in situ hybridization study with 35S-labeled MMP-1, reported an increased MMP-1 gene expression in the fibrous capsules of PTC but not in the cancer cells [21]. They considered this finding to be related to MMP-1’s role in the invasion of thyroid cancer [21]. When correlating MMP-1 expression between our two PTC groups, the invasive tumor group presented with statistically significantly increased MMP-1 expression in tumor cells (80.0% (67.5–90.0%) vs. 70.0% (45.0–85.0%); *p* = 0.049) and in adjacent non-tumorous-tissue cells (60.0% (50.0–80.0%) vs. 50.0% (37.5–65.0%); *p* = 0.029) of invasive PTC. This finding in our study further supports MMP-1’s role in PTC progression.

MMP-2 expression is reported to be significantly correlated with PTC, regardless of the metastatic status. Liang et al. reported similarly high immunohistochemical expression of MMP-2 and MMP-9 in metastatic PTC, suggesting that the expression of MMP-2 and MMP-9 may persist in the metastatic stages of PTC [23]. Tan et al. reported increased MMP-2 expression in tumor tissues with extrathyroidal metastases [24]. Many other studies have reported a correlation between high serum levels of MMP-2, PTC nodal metastases, and worse prognosis [23,24,25,26,27,28,29,30,31]. Contrary to these findings, Cavalheiro et al. found no correlation between MMP-2 immunohistochemical expression and the presence of nodal metastases in PTC tissue [25]. In our study we do not report any statistically significant difference in MMP-2 expression among our observed PTC groups. We attribute this finding to differences in methodology and the fact that our study only focused on invasive and non-invasive PTC, whereas previous studies compared malignant and benign disease and made no distinction in the level of invasiveness.

When reviewing the literature on the observed expression of MMP-9 in PTC, we found that Marečko et al. reported an increased immunoexpression of active MMP-9 in PTC and its positive correlation with lymph node metastases, extrathyroid invasion, and the degree of neoplastic infiltration [31]. Liu et al. revealed that MMP-9 was overexpressed in PTC tissues when compared with benign thyroid nodule tissues [32]. Furthermore, MMP-9 scores yielded an area under the curve (AUC) of 0.842 (95% CI, 0.776–0.908) for differentially diagnosing PTC from benign thyroid nodules. In addition, the MMP-9 score was greater if patients previously had central lymph node metastasis, lateral lymph node metastasis, or an advanced tumor-node metastasis stage (III + IV) [32]. In addition to cervical lymph node metastases, Maeta et al. reported increased expression of MMP-9 with increasing tumor size, higher stage, and capsule invasion [30].

We report an increased MMP-9 expression in PTC, but contrary to the previous studies, we did not find any statistically significant correlation between MMP-9 expression in invasive and non-invasive PTC tissue. However, we do report statistically significantly increased MMP-9 expression in the adjacent non-tumor tissue of invasive tumors (82.5% (67.5–90.0%) vs. 70.0% (50.0–90.0%); *p* = 0.017) when correlated with the adjacent tissue of non-invasive PTC. This result further supports MMP-9’s role in PTC progression and its potential role as a prognostic marker for PTC.

Few studies have investigated the expression of TIMP-1 and TIMP-2 in PTC [20,28,30,34,35,36,37,38,39] and their results are contradictory. Several of these studies reported increased expression while others reported reduced TIMP expression in malignant tumors. Increased TIMP-1 expression was reported to correlate with tumor size, metastases, and stage of disease in PTC [20,28,30,34]. In contrast, Baldini et al. reported a reduced TIMP-1 expression in recurrent PTC [37].

In our study we report an increased TIMP-1 expression in tumor cells (95.0% (85.0–95.0%) vs. 82.5% (50.0–90.0%); *p* < 0.001) and significantly increased expression of TIMP-1 in the adjacent non-tumorous-tissue cells (80.0% (67.5–90.0%) vs. 50.0% (23.8–70.0%); *p* < 0.001) in invasive PTC when compared with non-invasive PTC.

Bumber et al. did not find any statistically significant difference in TIMP-2 expression between non-metastatic and metastatic PTC [20], which is in line with our study results, as we also report no statistically significant difference in TIMP-2 expression between invasive and non-invasive PTC. However, we did find an increased expression of TIMP-2 in invasive PTC.

When correlating MMP-1, MMP-2, MMP-9, TIMP-1, and TIMP-2 expression in tumor tissue in non-invasive and invasive PTC, we report significant positive correlation between TIMP-2 and MMP-2 in the non-invasive tumor group (rho = 0.378, *p* = 0.039). In the invasive tumor group we found two significant positive correlations: first between MMP-1 and MMP-2 expression (rho = 0.306, *p* = 0.031) and second between TIMP-2 and MMP-9 expression (rho = 0.464, *p* = 0.001).

This positive correlation of TIMP-2 expression with MMP-2 and MMP-9 in PTC could be explained by the fact that TIMP-2 is the most widely distributed metalloproteinase inhibitor that regulates MMP-2 and MMP-9 activity. MMP-9 expression was increased in tumor tissue of invasive PTC with a statistically significant increase of expression in the adjacent non-tumorous tissue of invasive PTC in our study.

Our research was focused exclusively on invasive and non-invasive PTC and the obtained results might differ if we include regional metastases or other possible and reported predictive factors for PTC, which is a limitation of our study. Furthermore, our study and control groups are small, and we included only patients who underwent total thyroidectomy at our own institution, thus presenting only limited information that may not be readily generalized to the entire population.

In conclusion, elevated expression of MMP-1 and TIMP-1 in tumor tissue can predict invasiveness for PTC. MMP-9 and TIMP-2 expression in invasive PTC are increased and, although the difference was not statistically significant in our study, we consider this finding of prognostic value. Future research on the identification of specific MMP targets at different stages of tumor progression could help tailor targeted therapy for this group of PTC with unfavorable prognoses.

## Figures and Tables

**Table 1:** Differences in histology type, T status, and patient gender distribution between non-invasive and invasive tumor types: Fisher–Freeman–Halton’s exact tests.

		Tumor Type				*p*
		Non-Invasive N = 30		Invasive N = 50		
		N	%	N	%	
Histology variant	Classic	24	80.0%	38	76.0%	0.741
	Follicular	4	13.3%	5	10.0%	
	Tall-cell	1	3.3%	1	2.0%	
	Solid	0	0.0%	1	2.0%	
	Oncocyte	1	3.3%	0	0.0%	
	Warthin-like	0	0.0%	1	2.0%	
	Clear cell	0	0.0%	1	2.0%	
	Diffuse sclerosing	0	0.0%	3	6.0%	
T grade	1a	7	23.3%	7	14.0%	0.588
	1b	3	10.0%	7	14.0%	
	2	2	6.7%	1	2.0%	
	3	18	60.0%	34	68.0%	
	4a	0	0.0%	1	2.0%	
Gender	Male	3	10.0%	15	30.0%	0.053
	Female	27	90.0%	35	70.0%	

**Table 2:** Differences in age, tumor size, MMP-1, MMP-2, MMP-9, TIMP-1, and TIMP-2 expression in tumor cells and corresponding adjacent tissue between non-invasive tumors (N = 30) and invasive tumors (N = 50): Mann–Whitney U tests.

Tumor Type		Min	Max	Percentiles			Mann–Whitney U	Z	*p*
				25th	Median	75th			
Age (years)	Non-invasive	15.0	79.0	41.8	54.0	62.3	527.0	−2.2	0.027
	Invasive	11.0	73.0	30.8	45.0	54.3			
Tumor size (cm)	Non-invasive	0.2	5.0	0.6	1.0	1.5	514.0	−2.4	0.018
	Invasive	0.4	7.6	1.0	1.2	2.0			
MMP-1 tumor	Non-invasive	0.0	90.0	45.0	70.0	85.0	554.0	−2.0	0.049
	Invasive	10.0	95.0	67.5	80.0	90.0			
MMP-2 tumor	Non-invasive	0.0	95.0	0.9	15.0	68.8	721.0	−0.3	0.772
	Invasive	0.0	90.0	5.0	20.0	32.5			
MMP-9 tumor	Non-invasive	0.0	90.0	30.0	70.0	90.0	569.0	−1.8	0.069
	Invasive	0.0	95.0	50.0	80.0	90.0			
TIMP-1 tumor	Non-invasive	0.0	95.0	50.0	82.5	90.0	411.0	−3.5	<0.001
	Invasive	0.0	95.0	85.0	95.0	95.0			
TIMP-2 tumor	Non-invasive	0.0	95.0	5.0	10.0	26.3	5678.0	−1.7	0.086
	Invasive	0.0	95.0	5.0	30.0	56.3			
MMP-1 adjacent tissue	Non-invasive	15.0	85.0	37.5	50.0	65.0	532.0	−2.2	0.029
	Invasive	10.0	90.0	50.0	60.0	80.0			
MMP-2 adjacent tissue	Non-invasive	0.0	75.0	10.0	30.0	70.0	736.0	−0.1	0.893
	Invasive	5.0	85.0	17.5	30.0	56.3			
MMP-9 adjacent tissue	Non-invasive	20.0	90.0	50.0	70.0	90.0	514.0	−2.4	0.017
	Invasive	20.0	95.0	67.5	82.5	90.0			
TIMP-1 adjacent tissue	Non-invasive	0.0	95.0	23.8	50.0	70.0	352.0	−4.0	<0.001
	Invasive	20.0	95.0	67.5	80.0	90.0			
TIMP-2 adjacent tissue	Non-invasive	0.0	90.0	5.0	20.0	42.5	622.0	−1.3	0.201
	Invasive	0.0	80.0	10.0	30.0	40.0			

**Table 3:** Multivariate prediction of belonging to the invasive tumor group: binary logistic regression.

	OR	95% CI		*p*
		Lower	Upper	
Age (years)	0.97	0.94	1.01	0.109
Tumor size (cm)	1.81	1.01	3.25	0.048
MMP-1 tumor	1.20	0.53	2.74	0.664
TIMP-1 tumor	1.36	0.70	2.63	0.359
MMP-1 tumor	1.37	0.50	3.76	0.537
MMP-9 adjacent tissue	0.73	0.32	1.69	0.460
TIMP-1 adjacent tissue	3.97	1.42	11.13	0.009

**Table 4:** Correlation between MMP-1, MMP-2, MMP-9, TIMP-1, and TIMP-2 expression in tumor tissue in non-invasive and invasive tumor types: Spearman correlation coefficients (rho).

Tumor Type			MMP1	MMP2	MMP9	TIMP1	TIMP2
Non-invasive N = 30	MMP-1	Correlation Coefficient	1.000	0.313	0.133	0.230	0.344
		*p*		0.093	0.484	0.221	0.062
	MMP-2	Correlation Coefficient	0.313	1.000	0.281	0.211	0.378
		*p*	0.093		0.132	0.263	0.039
	MMP-9	Correlation Coefficient	0.133	0.281	1.000	−0.051	0.115
		*p*	0.484	0.132		0.790	0.546
	TIMP-1	Correlation Coefficient	0.230	0.211	−0.051	1.000	0.353
		*p*	0.221	0.263	0.790		0.056
	TIMP-2	Correlation Coefficient	0.344	0.378	0.115	0.353	1.000
		*p*	0.062	0.039	0.546	0.056	
Invasive N = 50	MMP-1	Correlation Coefficient	1.000	0.306	−0.093	−0.056	−0.238
		*p*		0.031	0.522	0.699	0.095
	MMP-2	Correlation Coefficient	0.306	1.000	−0.019	−0.127	−0.081
		*p*	0.031		0.893	0.379	0.577
	MMP-9	Correlation Coefficient	−0.093	−0.019	1.000	0.145	0.464
		*p*	0.522	0.893		0.315	0.001
	TIMP-1	Correlation Coefficient	−0.056	−0.127	0.145	1.000	0.040
		*p*	0.699	0.379	0.315		0.785
	TIMP-2	Correlation Coefficient	−0.238	−0.081	0.464	0.040	1.000
		*p*	0.095	0.577	0.001	0.785	

## Data Availability

The data that support the findings of this study are not publicly available because they contain information that could compromise the privacy of research participants. They can be made available on request to the corresponding author.
